# New insights into the spatial variability of microbial diversity and density in peatlands exposed to various electron acceptors with an emphasis on methanogenesis and CO_2_ fluxes

**DOI:** 10.3389/fmicb.2024.1468344

**Published:** 2024-10-15

**Authors:** Sadaf Shabbir, Chang Qian, Muhammad Faheem, Fengwu Zhou, Zhi-Guo Yu

**Affiliations:** ^1^School of Hydrology and Water Resources, Nanjing University of Information Science and Technology, Nanjing, China; ^2^Department of Agricultural Resources and Environment, College of Applied Meteorology, Nanjing University of Information Science and Technology, Nanjing, China; ^3^School of Geography, Nanjing Normal University, Nanjing, China

**Keywords:** TEAs, CH_4_ emission, *Methylomirabilales*, peatland mesocosms, *Methylococcales*

## Abstract

Peatlands are vital in the global carbon cycle, acting as significant sinks for carbon and releasing methane (CH_4_) and carbon dioxide (CO_2_) into the atmosphere. However, the complex interactions between environmental factors and the microbial communities responsible for these greenhouse gas emissions remain insufficiently understood. To address this knowledge gap, a pilot-scale mesocosm study was conducted to assess the impact of different terminal electron acceptors (TEAs), including sulfate (SO_4_^2−^), humic acid (HA), and goethite, on CH_4_ and CO_2_ emissions and microbial community structures in peatlands. Our results revealed that the addition of TEAs significantly altered the CH_4_ and CO_2_ emissions. Specifically, the addition of SO_4_^2−^ nearly doubled CO_2_ production while substantially inhibiting CH_4_ emissions. The combined addition of SO_4_^2−^ and HA, as well as HA alone, followed a similar pattern, albeit with less pronounced effects on CH_4_. Goethite addition resulted in the highest inhibition of CH_4_ among all treatments but did not significantly increase CO_2_ production. Community composition and network analysis indicated that TEAs primarily determined the structure of microbial communities, with each treatment exhibiting distinct taxa networks. *Proteobacteria*, *Acidobacteria*, *Chloroflexi,* and *Bacteroidetes* were the most abundant phyla across all mesocosms. The presence of methanotrophs, including *Methylomirabilales* and *Methylococcales*, was linked to the inhibition of CH_4_ emissions in these mesocosms. This study provides novel insights into the spatial variability of microbial diversity and density in peatlands under various TEAs, emphasizing the role of methanogenesis and CO_2_ fluxes in carbon cycling. Our findings enhance the understanding of carbon cycling in microbe-rich environments exposed to TEAs and highlight the potential for future studies to investigate the long-term effects of TEAs on microbial communities, enzymes, and carbon storage.

## Graphical abstract

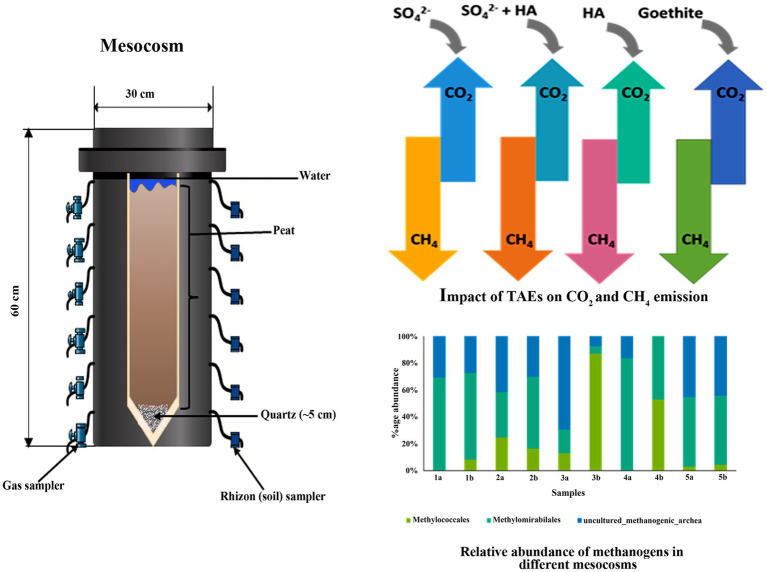

## Highlights

- Addition of TEAs to peatlands will affect the carbon cycle within the soil.
- There is a tendency for TEAs to suppress CH_4_ emissions and increase CO_2_ emissions.
- TEAs profoundly affect the structure and abundance of peatland microbiota.
- The methanotrophs *Methomylomirabilales*, and *Methomylococcales* were amplified by the addition of TEAs.

## 1 Introduction

Peatlands are well-known for their significance as carbon sinks among all terrestrial ecosystems due to the higher C reserves of 15–30% of soil organic C. Moreover, they are considered to sink to C by taking 20% of the total CO_2_ and emitting 5–25% of CH_4_ into the atmosphere (Frolking et al., 2011). According to Neubauer and Megonigal (2015), over 100 years, CH_4_ will have a more sustainable impact on global warming that is 45 times higher than CO_2_. Consequently, the ratio of anaerobic production of these two gasses, by decomposition of carbon, could result in drastic temperature changes in future. Therefore, it is imperative to understand the processes involved in converting stored carbon in peatland soils into CO_2_ and CH_4_ (Frolking and Roulet, 2007). Furthermore, the extent of impacting the environmental carbon cycle makes it essential to evaluate the impact of factors affecting their C cycle.

Due to global changes in the environment, several factors are responsible for the emission rate of greenhouse gasses, CO_2_ and CH_4_, from peatland soils, including temperature (Matysek et al., 2019), wildfires (Rodriguez Vasquez et al., 2021), land use (Tan et al., 2020), availability of nutrients (Shi et al., 2021), vegetation (Shirokova et al., 2021; van Lent et al., 2019), water table (Matysek et al., 2019; Shi et al., 2021), and oxygen (Girkin et al., 2020), etc. As a consequence of these environmental or anthropogenic changes, the emission of CO_2_ and CH_4_ may also increase from the carbon stock of peatland, which, in turn, also alters the microbial community structure in a peatland. Microbial communities of peatland play a crucial role in regulating carbon emission processes (Cong et al., 2020). Though in anaerobic methanogenic conditions, the amount of CH_4_ exceeds CO_2_, in some acidic bogs, anaerobic CO_2_ production exceeds CH_4_ (Hines et al., 2008; Keller and Bridgham, 2007) showing significant non-methanogenic activity of microbes. Though bacterial communities differ among different peatland types, anaerobic bacteria, along with providing substrates for methanogens, compete with the process of methanogenesis as well (Lin et al., 2012). Consequently, the role of environmental and ecological conditions along with microbial community structure is crucial in understanding the production of greenhouse gasses by these systems.

Heretofore, it has been reported that the dominant communities in peatlands are similar at certain taxonomic levels, e.g.*, Acidobacteria* and *Proteobacteria* being the dominant phyla. However, a variable correlation was found in environmental factors and microbial communities of peatlands (Andersen et al., 2013; Gilbert and Mitchell, 2006). Recently, many ecological elements have been reported to affect the microbial community structure of the peatlands, such as temperature (Keiser et al., 2019), pH (Bahram et al., 2018; Urbanová and Bárta, 2014), water table (Urbanová and Bárta, 2016; Zhong et al., 2017), nitrogen contents (Pankratov et al., 2008), phosphorus, moisture and organic matter (OM) (Elliott et al., 2015), etc. Moreover, Dhandapani et al. (2019) have reported that the abundance bacterial community in peatlands is positively correlated to the emission of CO_2_ and CH_4_. Thus, it is essential to determine which dominant microbial communities are responsible for the emission of greenhouse gasses (GHGs) from different peatlands under different circumstances. Therefore, studying microbial community structure and its variations is crucial in interpreting greenhouse gas dynamics. In water-logged anoxic conditions, the ratio of CH_4_ and CO_2_ is primarily affected by terminal electron acceptors (Gao et al., 2019). The carbon cycling in peatlands is mainly affected by the process of transferring electrons from electron donors to acceptors in peat (Walpen et al., 2018). Terminal electron acceptors (TEA) play a crucial role in microbial respiration, and microbes gain chemical energy by electron transfer between an electron donor and acceptor through oxidation of substrates. The production of CO_2_ is mediated by adding auxiliary TEAs, which increase the degradation of organic matter.

Moreover, it has already been reported that the addition of TEAs results in suppression of CH_4_ formation as it thermodynamically favors respiration more than methanogenesis (Bridgham et al., 2013). Peatlands have different types of redox conditions and have an abundant quantity of various nutrients readily available for metabolically active microbes and redox processes (Kügler et al., 2019). Several studies have already reported that humus (Valenzuela et al., 2019); nitrate (Haroon et al., 2013); Fe(III) (Cai et al., 2018; Ettwig et al., 2016), nitrite (Shen et al., 2017), Mn(IV) (Ettwig et al., 2016), and sulfate (SO_4_^2−^) (Shen et al., 2019) serve as TEAs for methanotrophs that consume a more significant part of CH_4_ produced in anoxic soil layers. Although several studies have described the variation in microbial community structure and production of greenhouse gasses in response to different factors such as inundation (de Jong et al., 2020) of peatlands, knowledge about the microbial community structure in response to redox reaction is not very clear. Therefore, understanding the impact of different TEAs on functional microbial communities and electron transfer mechanism resulting in the production of CH_4_ and CO_2_ is essential_._

The functional impact of environmental conditions and microbial community structure are studied in the present study. The primary objective of this research was to investigate CH_4_ and CO_2_ cycling as a function of soil depth and to evaluate the impact of humic acid, humic acid + sulfate, sulfate, and iron as TEAs on it. Peatlands are home to most of these TEAs. Furthermore, the response of the internal processes after the addition of TEAs on the community structure and abundance of microbial entities. Therefore, it was hypothesized that though the dominant phyla might be similar to other peatlands, TEAs will have an evident impact on microbial community structure. The findings of this study are expected to contribute to a better understanding of the environmental behavior of peatland soils, including emissions of carbon dioxide and methane gasses, as well as the composition of microbial communities following the addition of TEAs.

## 2 Materials and methods

### 2.1 Site description

**Figure 1:**
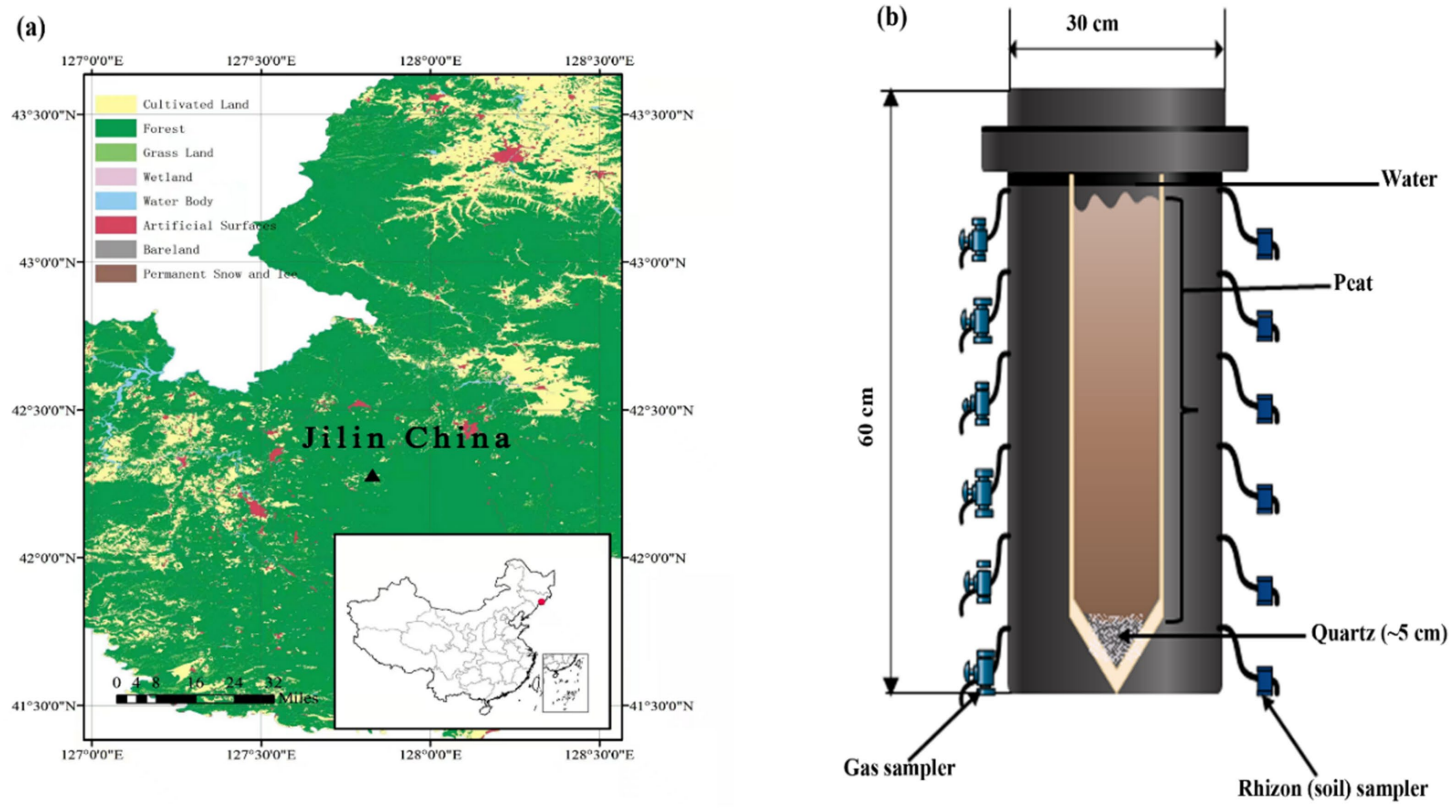
**(a)** Sampling site (Digital Map are derived from the Standard map services provided by the Ministry of Natural Resources of China; https://www.gscloud.cn) and **(b)** schematic illustration of the experimental design.

The soil was collected from a raised bog, Touxi (42°17.144 N, 127°50.277E, as indicated by the triangle; Figure 1a), located northwest of the dormant volcano found in the Changbai Mountain range situated at the boundary between northeastern China and North Korea. Topographically, the area is miscellaneous, comprising hills, steep slopes, valleys, and hills. The average annual temperature is around −7 to +3°C. The mean annual precipitation of 700–1,400 mm, and the peatland vegetation was mainly dominated by *Sphagnum* sp. along with a mixture of *Sanguisorba* sp., Carex, *Thelypteris* sp., *Iris* sp., *Aulacomnium palustre*, *Orchis* sp., *Eriophorum angustifolium*, *Oxycoccus palustris*, *Trichophorum* sp. and *Euphorbia* sp. The sample was collected up to 50 cm in depth, sealed in polyethylene plastic bags, and stored at 0°C before further analysis at the laboratory.

### 2.2 Mesocosm experiment

The sample was thawed to room temperature in the laboratory, and all of the peat was milled into small particles (diameter < 1 cm) manually. Then, it was homogenized to ensure an equal amount of organic matter in the whole sample, and plant roots and debris were removed. A subsample was collected at the start and was frozen at −20°C for DNA analysis within a week.

The mesocosms experimental units were polyvinyl chloride cylinders that were 60 cm high and 30 cm wide (Figure 1b) and were placed in the greenhouse to maintain a constant temperature (25°C). Each column was filled with clean quartz at the bottom with a thickness of ~5 cm prior to peat addition, then fine white mesh was plated to isolate the next added peat and quartz. Subsequently, native soil (peat) was added with a total depth of ~50 cm, i.e., about 5 cm below the top of the column. The mesocosm was then filled with water, and the water table was kept 3 cm above the surface of the peat in order to avoid O_2_ diffusion from air. Additionally, small pieces of PE shading foil were added to surface water to prevent algal growth. After about 65 days, the concentration of CO_2_ and CH_4_ have reached a steady state. Therefore, each of the samples, except control, was amended for the following treatments (1) Control (just with sand and peat-filled column and water); (2) Addition of 0.03 mol SO_4_^2−^ (Na_2_SO_4_); (3) Addition of 0.03 mol SO_4_^2−^ + 0.5 g HA; (4) Addition of generally 0.5 g HA and (5) Addition of 0.05 mol (generally) Goethite (as a source of Fe). Each mesocosm was also equipped with water and gas samplers. Pore water and silicon gas samplers were installed at 5, 10, 15, 25, 35, and 45 cm depth, as shown in Figure 1b. The mesocosms were allowed to settle for the first 60 days and then incubated in the laboratory for ~223 days (in total) in a climate chamber at 15°C (16 h light/8 h dark cycles, 660 μmol s^−1^ photosynthetic photon flux). Along with measuring CO_2_ and CH_4_ at different depths, the flux of these gasses was also measured on similar days (68, 75, 85, 96, 103, 116, 129, 143, 157, 181, 195, 207, and 223 days).

### 2.3 Sample analysis and calculations

Gas samples were collected, and dark chamber flux measurements were conducted on days 68, 75, 85, 96, 103, 116, 129, 143, 157, 181, 195, 207, and 223 days. Compared to the values obtained at different depths of mesocosm designated as the control, the percentage values have been calculated and given along with actual values in the results. The samples were analyzed to measure the amount of CH_4_ and CO_2_ were measured using a gas chromatograph (Agilent 7890A, Japan) equipped with FID and a CO_2_ methanizer.

Additionally, a vented stated chamber was used to collect surface gas samples from each mesocosm. CH_4_ and CO_2_ fluxes were measured on similar dates for each mesocosm. A dark chamber (20 cm x 21 cm) was used to determine the flux of these gasses through dynamic chamber measurement. Briefly, three identical floating chambers were placed on the top of mesocosms to estimate a better representative measurement as GHG emission on the water surface is heterogeneous. Fluxes from the headspace were calculated by linear regression of at least six consecutive readings within 30 min (one reading every 5 min) and stored for further analysis. A long measurement period was used to detect even the most minor changes in GHG fluxes. The concentration was later measured by the same method in GC as described above.

### 2.4 DNA extraction and real-time PCR

For DNA analysis, samples were collected from two places, the subsurface layer, and the bottom layer. The samples were named accordingly (Table 1). E.Z.N.A.^®^ DNA Kit for Soil (Omega Bio-tek, Norcross, GA, United States) was used to extract the microbial DNA from all mesocosms soil samples, following the given manual. 2% agarose gel was used to evaluate the quality of the DNA. The V4-V5 region of the bacteria 16S ribosomal RNA gene was amplified by PCR using forward primer 515F (5′-barcode-GTGCCAGCMGCCGCGG-3′) and 907R (5′-CCGTCAATTCMTTTRAGTTT-3′). The barcode is a unique eight base sequence for each sample. PCR mixture included of 20 μL mixture comprising four μL of 5 × FastPfu Buffer, 2 μL of 2.5 mM dNTPs, 0.8 μL of each primer (5 μM), 0.4 μL of FastPfu Polymerase, and 10 ng of template DNA. Agarose gel (2%) was used to extract amplicons that were purified by using AxyPrep DNA Gel Extraction Kit (Axygen Biosciences, Union City, CA, United States) according to the manufacturer’s instructions and later Quantus^™^ Fluorometer (Promega, United States) was used to quantify them.

**Table 1:** Sampling points in mesocosms and abbreviations for DNA analysis (a is the sample taken from the surface layer sample, and b is the sample taken from the bottom layers).

Mesocosms number	TEAs used	Abbreviations
1	Control	1a
		1b
2	SO_4_^2−^	2a
		2b
3	SO_4_^2−^ + HA	3a
		3b
4	HA	4a
		4b
5	Goethite	5a
		5b

The PCR products were quantified using Qubit^®^3.0 (Life Invitrogen), and amplicons (every 24) with different barcodes were equally mixed. In addition, Illumina’s genomic DNA library preparation procedure was used to construct the Illumina Pair-End library from pooled DNA at Shanghai BIOZERON Biotechnology Co., Ltd. (Shanghai, China) on the NovaSeq PE250 platform.

#### 2.4.1 Data processing

Raw fastq files were demultiplexed, quality filters using fastp version 0.20.0 (Chen et al., 2018), and the 300-bp reads were truncated at any site with an average quality score < 20 over a 50-bp sliding window. The truncated reads shorter than 50 bp were discarded, and the reads with ambiguous characters were discarded as well (Ren et al., 2016). Additionally, the sequences over 10 bp were assembled, discarding the rest. Later, the sequences were merged and quality filtered using FLASH version 1.2.11 (Magoč and Salzberg, 2011) and fastp version 0.19.6 (Chen et al., 2018). Then, the high-quality sequences were denoised using the DADA2 (Callahan et al., 2016) plugin in the Qiime2 version 2020.2 (Bolyen et al., 2019) package with suggested parameters filtering nucleotides based on error profiles within samples, and the resulting denoised samples are known as amplicon sequence variants (ASVs). Briefly, the 119, 009 raw reads were trimmed by quality control and filtering contaminates to create ASV with 109, 572 sequences with approximately 99.2% good coverage. Finally, the QIIME2 feature-classifier plugin (sklearn method) against the SILVA database (version 138; trimmed to the V4-V5 version of the 16 S) was used to determine the taxonomic identities of the ASVs. QIIME 2 was used to calculate different statistical measures from the ASV table that was normalized to 20,000 sequences per sample. The relevant resources are available at: https://github.com/LangilleLab/microbiome_helper/wiki.

### 2.5 Statistical analysis

In order to analyze ASV files obtained from QIIME 2, a microbiome analyst was used, which compiles and analyzes microbiome data online comprehensively and visually (Chong et al., 2020). Briefly, the alpha diversity (diversity of species composition) was determined by Shannon, ACE, Simpson, Chao1 index, and Fisher curves. In contrast, An analysis of similarities (ANOSIM) test was used to compare beta-diversity determined using Bray-Curtis distances and principal coordinate analysis plots (PCoA) based on Bray-Curtis distances. A heatmap of the most abundant classes was generated based on complete hierarchical clustering using Euclidian distances. Bacteria differentially represented in different mesocosms were evaluated by using linear discriminant analysis (LDA) together with effect size (LEfSe). A taxonomic cladogram illustrates differences among genera based on significant taxa (Segata et al., 2011).

## 3 Results

### 3.1 Impact of TEAs on CO_2_ and CH_4_ emission

**Figure 2:**
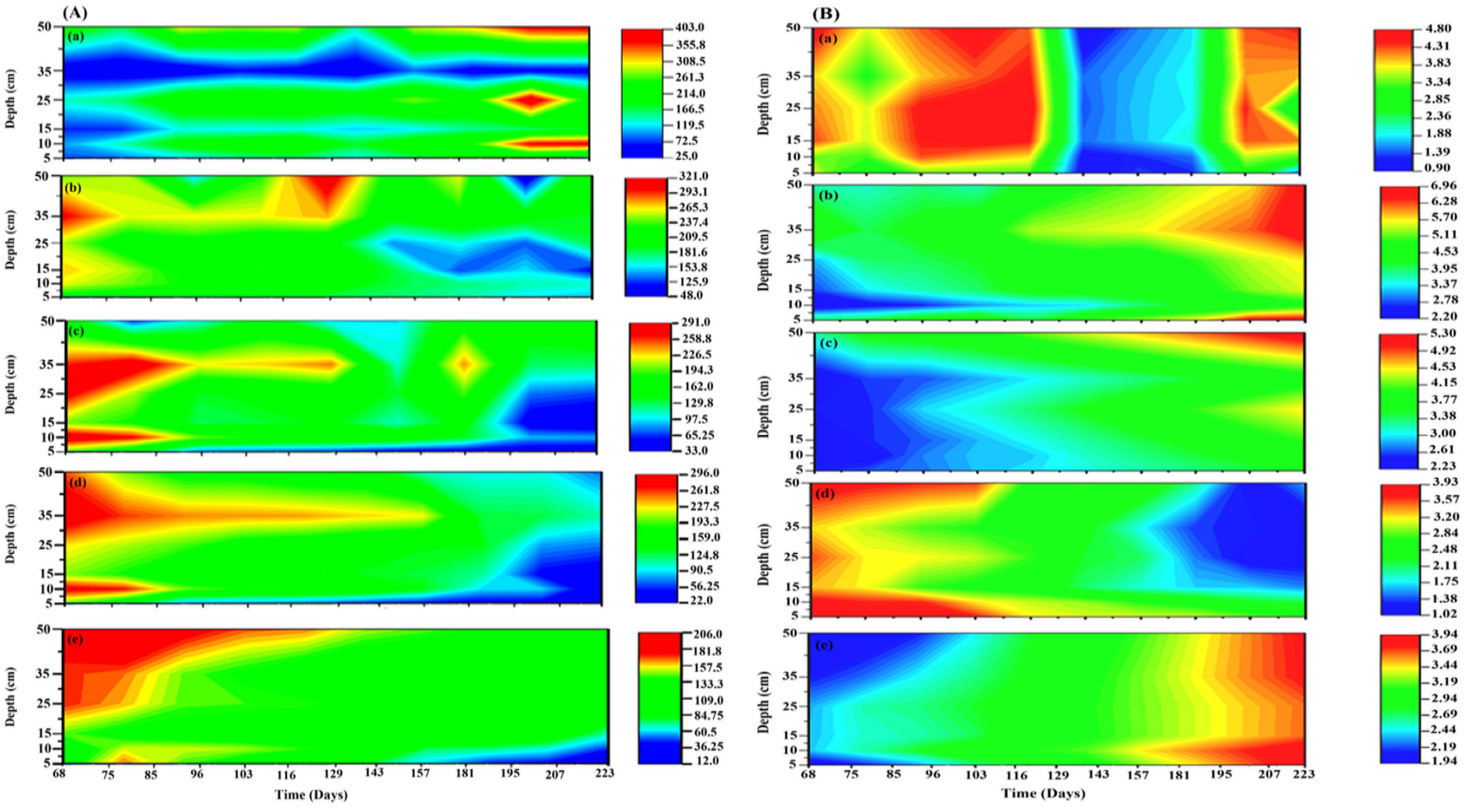
The concentration of **(A)** CH_4_ and **(B)** CO_2_ in 5 different mesocosms (a) control, (b) SO_4_^2−^; (c) SO_4_^2−^ + HA; (d) HA, and (e) Goethite. CH_4_ concentration was measured in μmol L^−1^, and CO_2_ concentration was measured in mmol L^−1^.

In the present study, four different treatments with different TEAs, (1) SO_4_^2−^; (2) SO_4_^2−^ + HA; (3) HA, and (4) Goethite, along with control, were studied to evaluate the impact of TEAs on CO_2_ and CH_4_ production as well as the density and diversity of microbial communities in peatland soils. In the first and second mesocosm, SO_4_^2−^ was added individually and with HA, respectively. The addition of SO_4_^2−^ resulted in higher depletion of CH_4_ in the surface layers of mesocosm, it was decreased up to 124.7 (↓56.2%) and 108 (↓ 71.2%) μmol L^−1^ in the 5 and 10 cm sample, respectively. In contrast, increased concentrations of sulfate were less detrimental to the production of CH_4_ (↓34%) in the deeper layers of the sediment (Figure 2Ab). The addition of SO_4_^2−^, however, led to an increase in the production of CO_2_ (Figure 2Bb). The addition of SO_4_^2−^ had increased CO_2_ by almost twice the amount of CO_2_ produced in control, i.e., 6.75–6.668 mmol L^−1^ (↑70–86.3%). After the addition of SO_4_^2−^ and HA, the production of CH_4_ was strongly inhibited, up to 36.06 μmol L^−1^ (↓90.49%) in the surface layers and ↓47.53% in the bottom layer. On the other hand, only a slight increase in CO_2_ production was observed between 3.55 and 5.29 mmol L-1 (↑18.18–24.7%) (Figures 2Ac,Bc). The CH_4_ production decreased significantly after HA was added, down to 56.06 μmol L^−1^ (↓55.9%) in the top layer and 127.7 μmol L^−1^ (↓41.42%) in the bottom layer. As with other TEAs, the addition of HA increased the production of CO_2_ following its addition from 29.5–46.6% (Figure 2Bd).

A drastic reduction in CH_4_ production was observed upon the addition of Goethite to 12.38 μmol L^−1^ (↓90.25%) in the top layer and 109.1 μmol L^−1^ (↓71.6%) in the bottom layer (Figure 2Ae), which was the highest reduction among all mesocosms. Interestingly as compared to CH_4_, goethite addition did not result in a significant increase in CO_2_ production, where its value was approximately 3.8 mmol L^−1^ (↑ ~ 19%) (Figure 2Be).

There were similar trends in surface-emissions at the top as in deeper soils, though the changes were more evident in top layers compared to the bottom layers of mesocosms. Briefly, the addition of TEAs inhibited and enhanced the emission of CH_4_ and CO_2_, respectively, is similar as explained for mesocosm 1–5 in deeper layers (Supplementary Figure S1).

### 3.2 Peat microbial communities

During lab-scale mesocosm experiments, gas emission varies from top to bottom, therefore, for DNA analysis the samples were taken at two different depths, from the top and near the bottom. A comparison was performed between different treatments based on the microbial diversity and community differences in mesocosms treated with different TEAs. After quality trimming and subsampling, the total number of high-quality reads generated by all the mesocosm samples was 116,231 (7,105–15,603 reads per sample, Supplementary Figure S2). As a result of taxonomic categorization at various levels, 28 phyla, 62 classes, 107 orders, 137 families, and 585 genera were identified.

**Figure 3:**
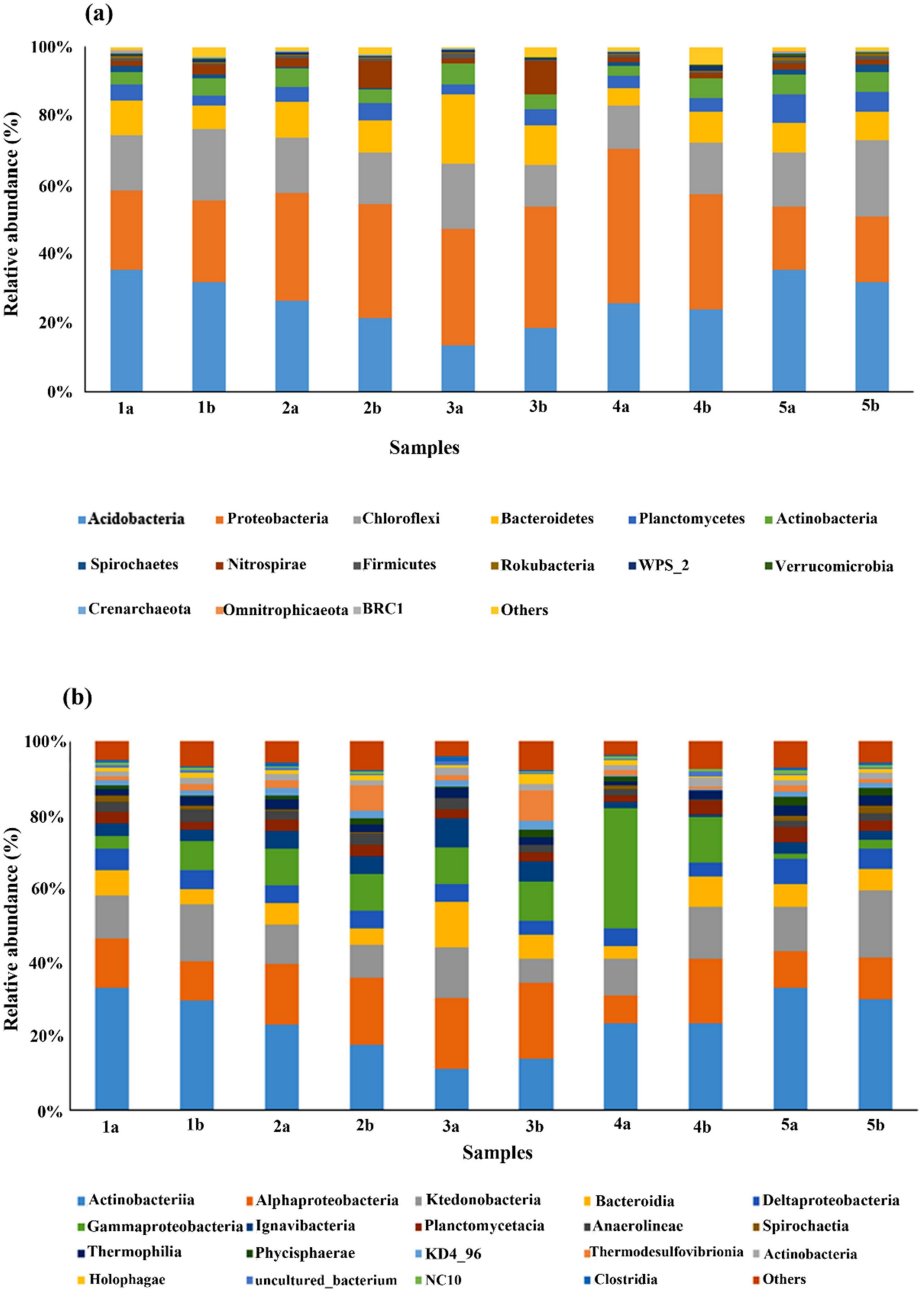
Comparison of microbial community structure in different mesocosms at **(a)** phylum level and **(b)** class level. There are two samples from each mesocosm, one was taken from surface layers (1), and the other was from the bottom layer (2) of each mesocosm.

Among all the 28 bacterial and archaeal phyla found in all the depths, bacterial phyla comprised ~90–97% of the total sequences. Among bacterial phyla, *Proteobacteria*, *Acidobacteria*, *Chloroflexi,* and *Bacteroidetes* were the most dominant phyla compared to others (Figure 3a). The most abundant phyla in the control group were *Acidobacteria* (11.76–33.13%), as well as in the HA treated group. *Proteobacteria* (18.3–44.3%) was the most abundant phyla in all the other treatments, followed by *Acidobacteria*. Soil-dwelling *Proteobacteria* play an essential role in the biogeochemical cycle. *Chloroflexi* (11.50–21.90%) and *Bacteroidetes* (5.05–20.11%) followed the same abundance pattern as *Proteobacteria*.

Other dominant phyla included *Planctomycetes*, *Actinobacteria*, *Spirochetes*, *Nitrospirae,* and *Firmicutes,* etc., whereas the rest of the phyla were less than 1% in their abundance. Additionally, the abundance pattern of different phyla varies after the addition of different external sources as well as with depth. For example, the abundance of *Acidobacteria* was decreased in all treatments after the addition of external sources, whereas that of *Proteobacteria* and other phyla showed relatively opposite trends.

Furthermore, the abundance of *Acidobacteria* was slightly higher in the top layers of mesocosms compared to the bottom layers. However, in HA treatment, it was higher in the bottom compared to the top layer. The abundance of other phyla also followed the same pattern as *Acidobacteria* except in HA-treated mesocosm. Our analysis also indicated the presence of 62 classes. The comparison of the dataset among these classes showed that *Acidobacteriia* was again the most abundant class in control as well as treatment groups, followed by *Alphaproteobacteria* and *Ktenobacteria*, *Gammaproteobacteria*, *Bacteroidia*, *Deltaproteobacteria*, *Igvanibacteria*, *Planctomycetacia*, *Thermosulfovibrionia*, and *Thermoleophilia,* etc., however, the dominance varies individually in every treatment (Figure 3b).

**Figure 4:**
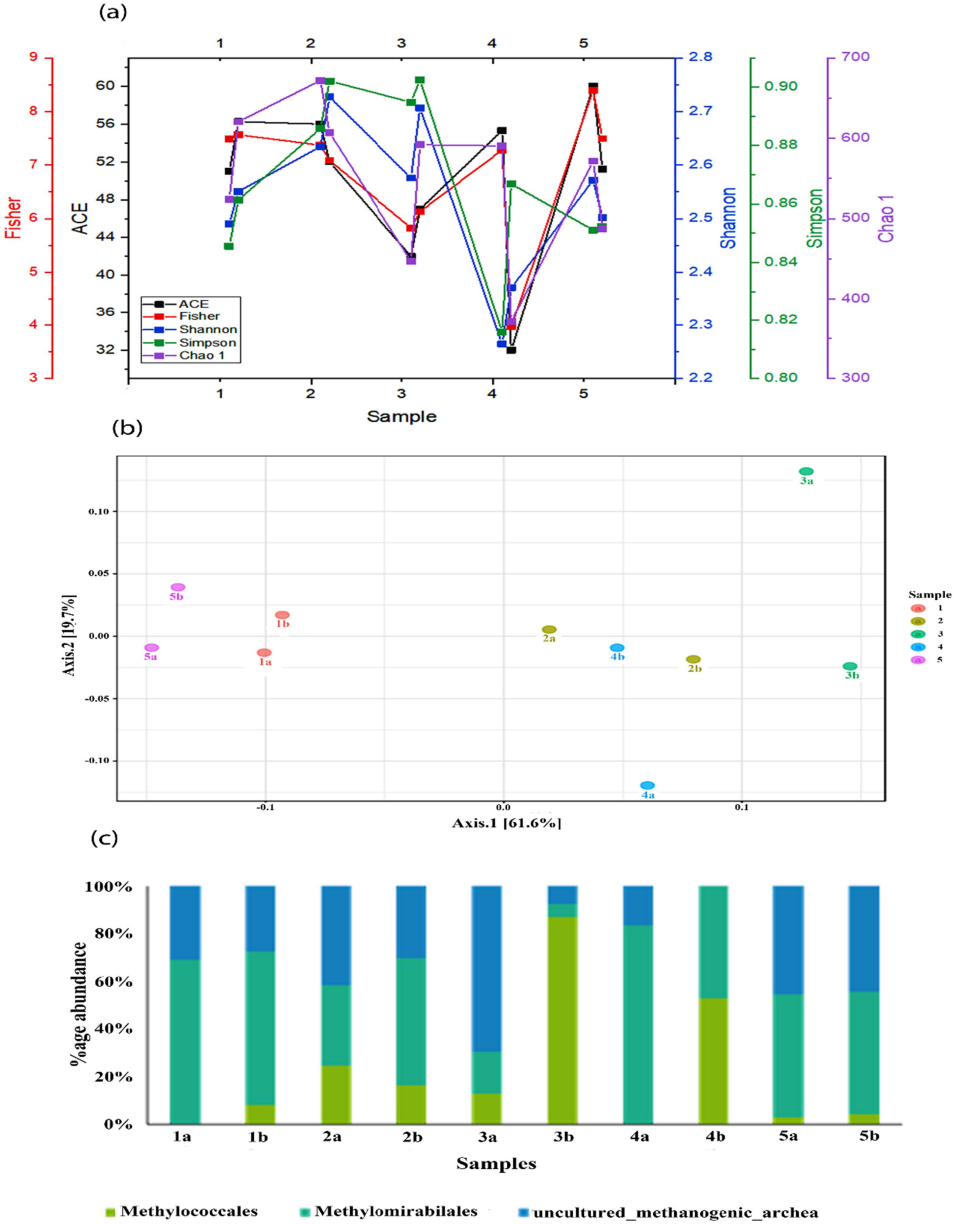
**(a)** Comparison of Shannon, Simpson, Chao1, and Fisher indices in different mesocosms; **(b)** principal coordinate analysis (PCoA) of different bacterial communities in mesocosms and **(c)** relative abundance of methanotrophs and methanogens found in different mesocosms.

As shown in Figure 4a, the alpha diversity indices, including Ace1, Fisher, Shannon, Simpson, and Chao showed variable trends. However, the HA treatment in all the treatments showed a decrease in the richness and diversity of the microbial community. Statistical analysis showed that alpha diversity was significantly lower in the treatment with HA alone than in the control group (*p* < 0.05). Additionally, the depth also affects the diversity indices, and the diversity decreased in all the mesocosms with the depth except after SO_4_^2−^ + 0.5 g HA addition.

#### 3.2.1 Methanotrophic and methanogenic communities

The main methantrophs found in this study were *Methylomirabilales*, *Methylococcales*, along with uncultured_methanogenic_archaeon (Figure 4b). The most abundant among these were *Methylomirabilales,* except in treatment with SO_4_^2−^ + 0.5 g HA. *Methylococcales* are type I methanotrophs and are also involved in nitrogen fixation, the second most abundant order in the mesocosms. Their abundance was substantially increased with the depth of mesocosm, which might be due to their sensitivity to oxygen; however, the highest increase was observed after the addition of SO_4_^2−^ + 0.5 g HA. Some uncultured methanogenic archaeon was also found in these mesocosms, and a subsequent decrease was observed in their abundance along with the depth.

Through visual inspection of principal coordinates (PCoA), we compared the results over time to determine the impact of different TEAs on the microbial community structure (Figure 4c). Class-level taxonomic classification between sample groups was taken into account as the experimental factor in the correlation analysis using the Pearson r distance measure. PCoA revealed a clear clustering pattern of microbial communities. PCoA 1 and PCoA 2 account for 61.6% and 19.7% of the total variance. Control mesocosm, without any external addition, differed from other mesocosms in terms of microbial community composition. Moreover, a clear difference can also be observed between the bottom and top microbial communities under the addition of TEAs.

#### 3.2.2 Heatmap and LEfSe analysis

**Figure 5:**
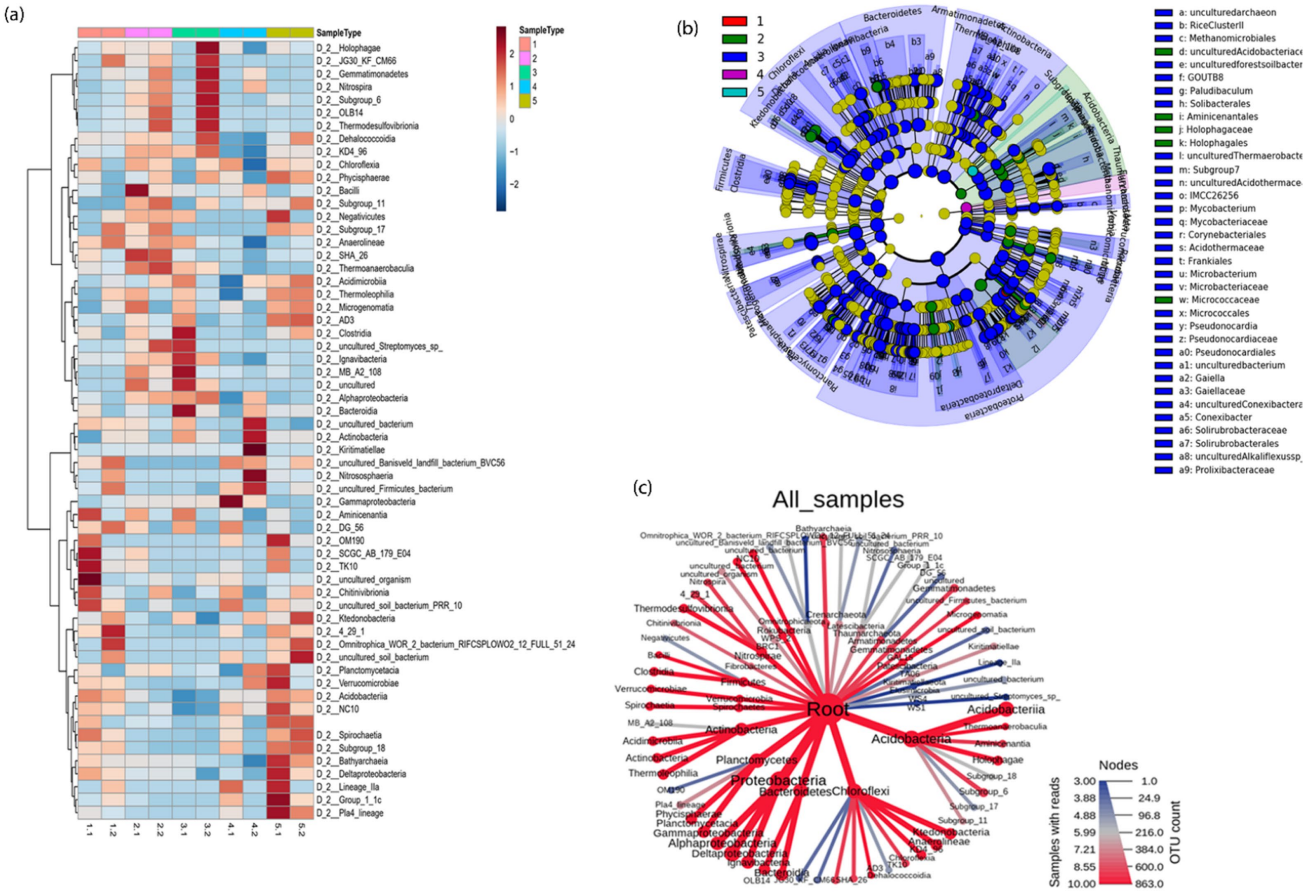
**(a)** Taxonomic differences in five mesocosms shown via a heat map; **(b)** A cladogram representing the structure of taxonomic hierarchies was derived using LEfSe to identify the significantly differentially abundant taxa in five mesocosms and **(c)** an overview of metacoder heatmaps for different mesocosms. Each node from the center (Kingdom) to outward (phylum) and then to classes.

The microbial community structure was further examined using a heatmap of the top 60 most prevalent microbial classes across the 10 samples. The relationships between microbial species and between the samples have been analyzed using cluster analyses (Figure 5a). In the tailings under different TEAs, there were substantial differences in the microbial composition at the class level.

The exact class-level changes of the bacterial community in different mesocosm are also given in above Figure 5c. The size of the node corresponds to the number of OTUs, and it can be seen that Acidobacteriia from phylum Acidobacteria was the most dominant one, followed by Proteobacteria and Bacteroidetes. We identified specific bacterial taxa under given conditions by comparing the linear discriminant analysis effect size (LEfSe) method to five mesocosm samples. The cladogram illustrates the relative abundance at the family and order levels in Figure 5b. Comparing mesocosms 1–5 (A, B, C, D, and E), 37 genera with LDA values of at least 3.5 were identified. LEfSe analysis showed that uncultured bacteria were most abundantly found in the control group (mesocosm 1); *Thaumarchaeota* was most abundant in mesocosm 2; *Proteobacteria*, *Bacteroidetes*, and *Chloroflexi* were most abundant in mesocosm 3, *Rhodocyclaceaebacterium*, *Betaproteobacterials,* and *Holophagales* were abundant in mesocosm 4 whereas TRA3_20 (uncultured *Betaproteobacteria*) were most abundant in mesocosm 5 compared to other mesocosms (Supplementary Figure S4). Using a cladogram (Figure 5b), we illustrate the connection between taxa at various taxonomic levels. Clades are groups of organisms that share a common ancestor, e.g., all Bacteroidetes are descendants of the same ancestor. Additionally, a significant difference in the abundance of different taxa might serve as a potential biomarker based on LDA results (LDA > 4).

## 4 Discussion

### 4.1 Repression of methanogenesis and higher CO_2_ production after TEAs addition

Oxygen (O_2_) has a higher oxidation capacity throughout natural ecosystems than redox ions such as SO_4_^2−^, Fe^3+^, and quinones of humic acid, giving precedence to redox reactions. Consequently, peat exposed to oxygen undergoes a faster redox reaction and decomposes more quickly. O_2_ is scarce in the lower layers of often flooded peatlands, and CO_2_ production is minimal (Osaki et al., 2021). The redox process creates CO_2_ as well as CH_4_ when the peat layer is strictly anaerobic. The response of different microbial metabolic activities in peatlands usually determines the ratio of CO_2_ and CH_4_ (CO_2_:CH_4_) production, which should be stoichiometrically 1:1 in the absence of any other TEAs (Wilson et al., 2017). However, a couple of laboratory scale and *in-situ* experimental studies have previously reported that this ratio is not the same, and CO_2_ production is enhanced by different environmental factors (Glaser et al., 2016). Therefore, It’s crucial to evaluate how different terminal electron acceptors (TEAs) influence CO2 and CH4 emissions, as studies exploring their combined effects are limited.

In the present study, the pilot scale mesocosms were used in the present study in order to simulate the natural peatland system as far as possible, with a larger incubator size close to natural status. It was observed that after the addition of SO_4_^2−^, a Pungent smell like H_2_S was felt in the first two mesocosms. It is important to note that this gas was not detected in deeper layers (results not shown here). The emission of H_2_S might be due to organoclastic sulfate reduction. It was observed that the addition of different TEAs has mostly inhibited the emission of CH_4_. However, the emission of CO_2_ increased. After the addition of SO_4_^2^, the reduction in CH_4_ production might be due to sulfur-reducing bacteria, which outcompete methanogens for peatland’s available carbon (Liu et al., 2020). Whereas the production of CO_2_ was doubled in this mesocosm, these results were in accordance with previous studies, where sulfate’s addition decreased methanogenesis and increased CO_2_ production (Estop-Aragonés et al., 2016; Minderlein and Blodau, 2010). The emission of CO_2_ and CH_4_ followed the same pattern in the third mesocosm, where sulfate was added in combination with HA.

The addition of HA had also impeded CH_4_ emission and enhanced CO_2_ production. One of the reasons for CH_4_ emission reduction might be the inhibitory effect of HA on methanogens, and previously it was reported that the addition of HA could decrease CH_4_ production by up to 89% (Khadem et al., 2017). Moreover, a significant part of humic acid’s function acts as an electron acceptor and constrains CH_4_ emissions. HA has a negative charge and electron shuttling properties, so a high concentration of HA inside methanogens might also alter their electron transport system. In comparison, HA serves as an electron acceptor for the reducing equivalents transported out of cells through cell membranes of methanogens due to alteration in the electron transport system suppressing microbial growth (Khadem et al., 2017). The peatland soils are generally rich in organic matter and are deficient in inorganic electron acceptors. As a result, the presence of HA would augment the process of microbial respiration, leading to an increase in CO_2_ production and deter CH_4_ production by suppressing methanogenic activity (Keller and Takagi, 2013; Klüpfel et al., 2014).

Goethite is basically hydrated iron oxide, and its addition showed similar trends to other TEAs in the present experiment. The presence of alternative TEAs such as Fe^3+^ and SO_4_^2−^ plays a vital role in deciding how the organic matter would be processed in a peatland. They are also reported to suppress methanogenesis, which is the primary form of anoxic mineralization in peatlands (Smemo and Yavitt, 2011). During water-logged conditions, the peatlands are O_2_ deficient, and the iron-reducing microorganisms utilize Fe as an electron acceptor and organic matter as an electron donor, resulting in the production of more CO_2_ (Kappler et al., 2021). Briefly, the addition of all the different TEAs increased CO_2_ production to some extent, which might have a constrained impact on the microbial communities involved in the respiration or methanogenesis process resulting in CO_2_ and CH_4_ production. Additionally, the minor changes in CO_2_ emission in the deeper layers can be related to the fact that microbial communities in the deeper layers are comparatively more resistant to external factors or nutrient deprivation (Bai et al., 2017a), the resistance against these changes results in the minor emission of CO_2_ in deeper layers (Liu et al., 2016) of mesocosm. Briefly, the addition of TEAs had significantly affected the amount CO_2_ and CH_4_ emission. Moreover, after TEAs addition, the stoichiometric 1:1 ratio between CH_4_ and CO_2_ was changed completely, sometimes doubling CO_2_ and/or suppressing CH_4_ more than 80%.

### 4.2 Addition of TEAs altered the microbial community structure

The microbial community structure differences were based on adding different TEAs. Nevertheless, the leading bacterial phyla were the same in all the mesocosm, but their abundance varied in the different mesocosm, *Proteobacteria* was more abundant in treatment 4a compared to control or 5a treatment. Similarly, *Bacteroidetes* abundance was much less in 4a compared to 3a (Figure 3a). Similar results were reported by Potter et al. (2017) that the proportion of microbial communities, especially, *Acidobacteria* and *Proteobacteria,* is significantly affected by the depth of peatland soils. The variation in the microbial community structure along depth is also due to oxygen deprivation in deeper layers. When oxygen is present in ample amounts, the aerobic microorganisms capable of methane oxidation are active (as in surface layers). Moreover, the aerobic environment, along with substrate addition, profoundly affects the community structure (Tian et al., 2022). The effect can also be seen in the previous section, where the addition of substrate has significantly affected gas emission in surface layers compared to deeper layers.

Additionally, the abundance results are also familiar with previous studies with the same bacterial phyla in different types of peatlands (Luo et al., 2021). Three microbial groups, *Bacteroidetes*, *Actinobacteria*, and *Verrucomicrobia*, were previously reported to contribute most to soil organic carbon degradation in northern peatlands (Tveit et al., 2013). Compared to this study, *Actinobacteria* and *Bacteroidetes* comprised a higher percentage compared to *Verrucomicrobia* in the present study. According to the present study, in peatland bacterial communities, the effects of TEAs appear to be rather subtle. Further investigations are needed to determine how these TEAs affect bacterial populations in specific ways. Due to their reliance on primary nutritional substrates for growth and metabolism, the microbial communities on the upper layers of the soil are more diverse.

The diversity indices also showed that the addition of TEAs has a profound impact on the density and diversity of microbial community structure (Figure 4a). Shannon diversity was comparatively higher in the top layer compared to the bottom layer, except for goethite addition which showed the opposite trends and vice versa. This might be due to physico-chemical changes in the properties of soil that are not feasible for the survival of the microbial communities, and only some bacterial strains survived that were able to grow in harsh climatic conditions (Zhang et al., 2019).

### 4.3 Relative abundance of methanotrophs and methanogens

According to the results, the main methanotrophs found in the present study were *Methylomirabilales*, *Methylococcales*, and uncultured_methanogenic_archaeon (Figure 4b). As the results showed, the most abundant of these were *Methylomirabilales* (Figure 4b). The microbial species in the order *Methylomirabilales* order can oxidize methane anaerobically as well as reduce nitrogen to dinitrogen (Ettwig et al., 2010). There is evidence that particulate methane monooxygenase (pMMO) encoding genes are present in the genomes of *Methylomirabilales* (Versantvoort et al., 2018). *Methylococcales* bacteria, primarily aerobic methane-oxidizing bacteria, are reported to be involved in AMO (anaerobic methane oxidation) (Cabrol et al., 2020). Briefly, an increase in microbial CH_4_ consumption is most likely behind the observed reduction in CH_4_ emissions under oxygen and other terminal electron acceptors.

### 4.4 LEfSe and heatmap analysis

The LEfSe analysis revealed the most abundant groups in 5 mesocosms were different from each other, While there is a limited amount of knowledge concerning the specific ecology of these groups in peatlands (Figure 5b). It is well documented that *Thaumarchaeota* species are found in soil samples worldwide, and their contribution to global biogeochemical cycles is crucial (Lin et al., 2015). In addition to having mixotrophic, chemoautotrophic, and heterotrophic lifestyles, *Thaumarchaeota* is capable of producing energy through diverse mechanisms. Peat biotopes are home to *Thaumarchaeota* where they produce living compounds by oxidizing organic fatty acids, amino acids, and inorganic ammonia. However, there is still uncertainty regarding the environmental role of *Thaumarchaeota* in peatlands. *Proteobacteria*, *Bacteroidetes,* and *Chloroflexi*, the most abundant groups in mesocosm 3, were also found in Northern peat lands. It has already been reported that a large proportion of peatlands throughout northern and tropical regions were dominated by *Proteobacteria* while *Bacteroidetes* and *Chloroflexi* constituted secondary dominant phyla (Finn et al., 2020). *Rhodocyclaceaebacterium*, *Betaproteobacterials,* and *Holophagales* were found in mesocosm 4. There are few available reports about detecting *Rhodocyclaceaebacterium*, *Betaproteobacterials*, and *Holophagales*, especially in peatlands. However, there has been evidence that *Betaproteobacterials* methylotrophs can utilize and assimilate ^13^C-methanol (Osaka et al., 2006). Whereas TRA3_20 (uncultured *Betaproteobacteria*) was abundant in mesocosm 5, and the information available about TRA3_20 is also limited.

## 5 Conclusion

The present study studied the impact of different TEAs on the emission of greenhouse gasses from peatland soils in pilot-scale mesocosms. It has been found that most TEAs have inhibited the production of CH_4_ and enhanced the production of CO_2_ to a certain extent. Proteobacteria, Acidobacteria, Chloroflexi, and Bacteroidetes were the most abundant phyla in all the mesocosms, and their dominance varied among different treatments. Additionally, the presence of methanotrophs, including *Methylomirabilales* and *Methylococcales,* might be the reason behind the decrease in the CH_4_ emission in different mesocosms due to its consumption by the following microorganism. We concluded that introducing a new electron acceptor to peatlands is likely to alter microbial community structure and diversity, which in turn could significantly influence CH_4_ and CO_2_ emissions. Future research should look at additional possible electron acceptors and related active microorganisms to understand better the global geochemical cycle and how methane and CO_2_ emissions are regulated in peatland soils.

## Data Availability

The datasets presented in this study can be found in online repositories. The names of the repository/repositories and accession number(s) can be found at: https://www.ncbi.nlm.nih.gov/search/all/?term=PRJNA1169397.
