# Supplementary material for: New insights into the spatial variability of microbial diversity and density in peatlands exposed to various electron acceptors with an emphasis on methanogenesis and CO2 fluxes
Source: Front Microbiol. 2024 Oct 15;15:1468344. doi: 10.3389/fmicb.2024.1468344 (PMC11520324; doi:10.3389/fmicb.2024.1468344)
Supplement: Supplementary file 1 [file Data_Sheet_1.PDF]

Supplementary Material

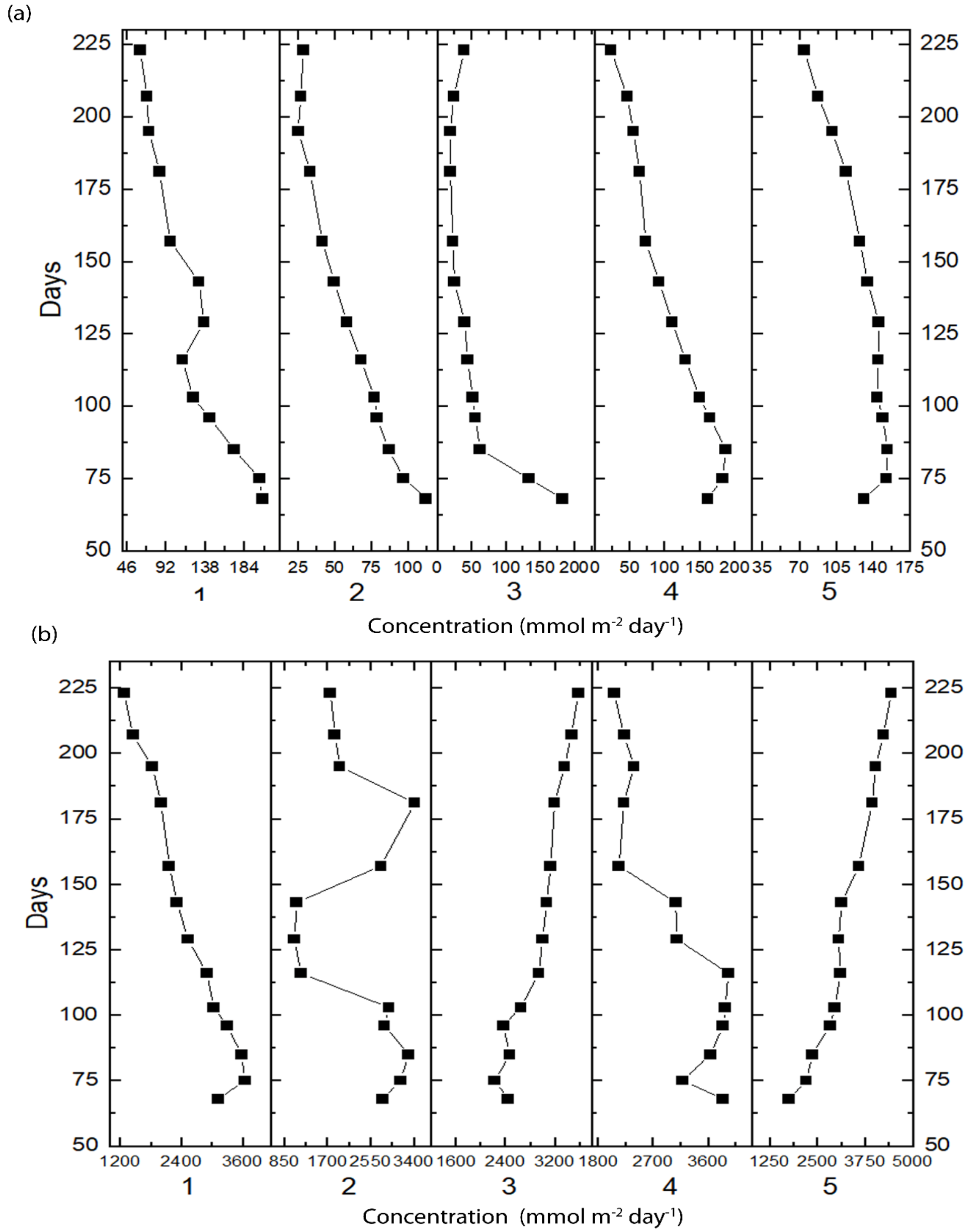

Fig. S1 Flux of GHGs (a)CO_2 and (b) CH_4 on the surface of 5 mesocosms at different time intervals

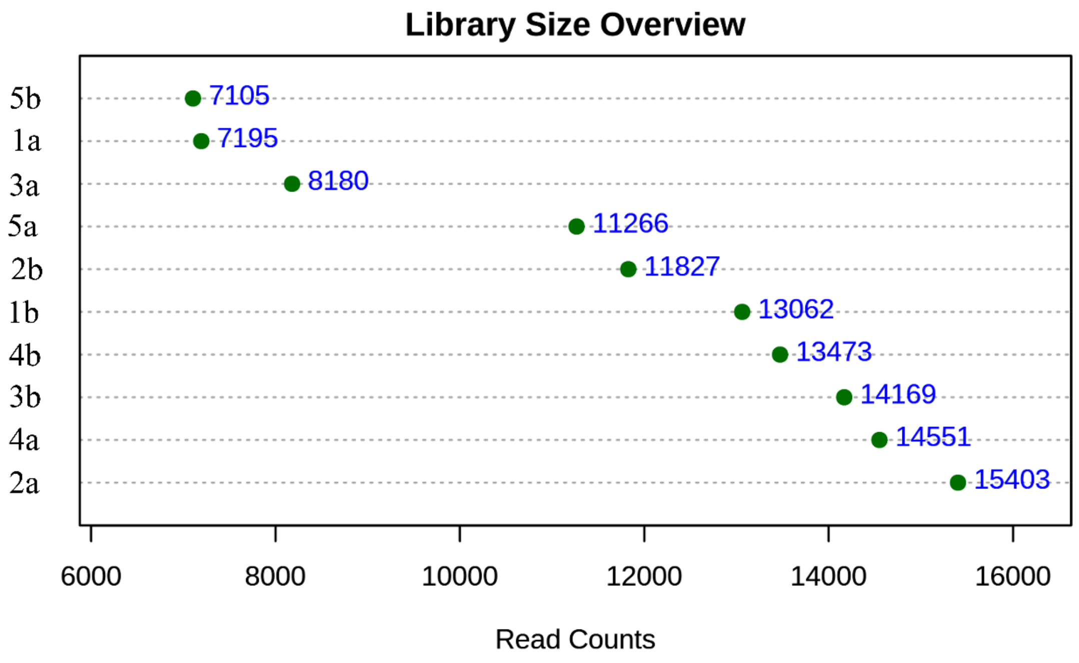

Fig. S2. High quality reads for each sample after quality trimming and subsampling

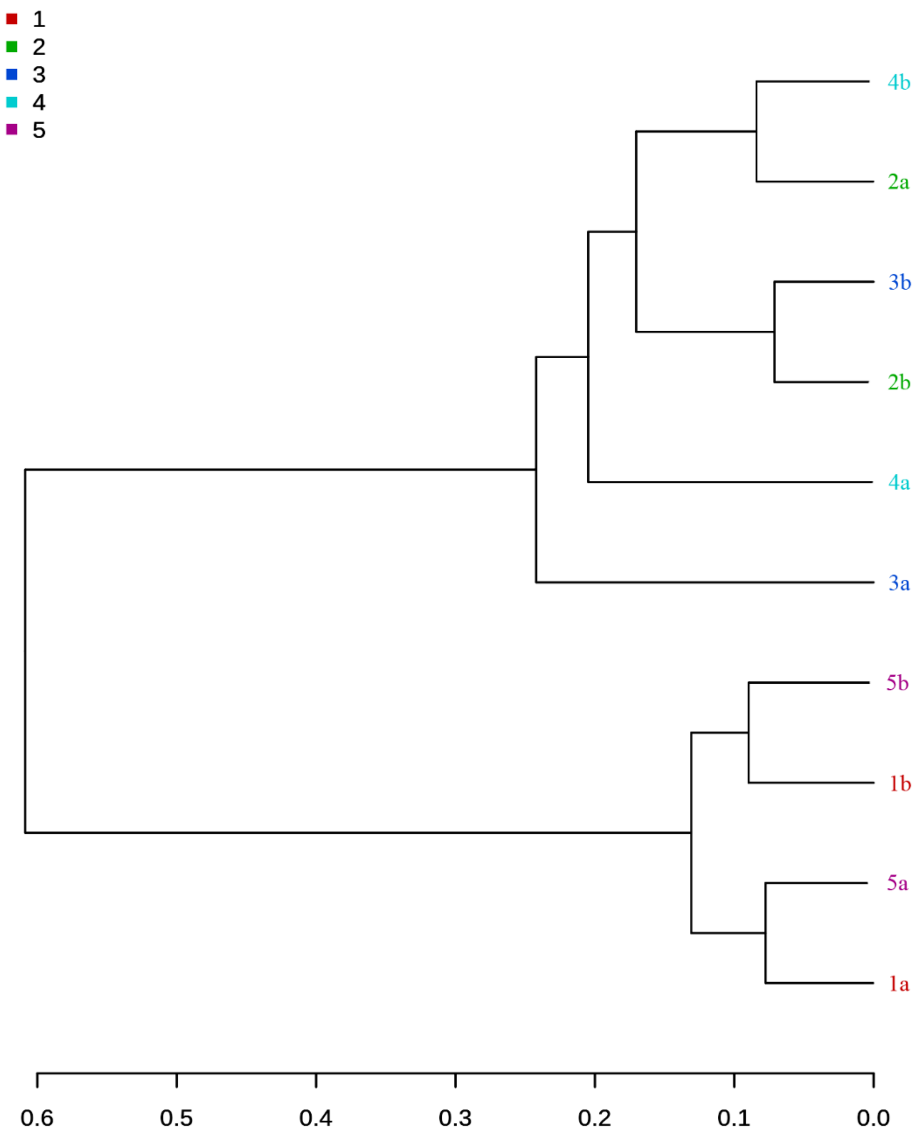

Fig. S3. Hierarchical cluster dendrogram of microbial communities present in surface and bottom layers of 5 different mesocosms based on Pairwise Bray-Curtis dissimilarity

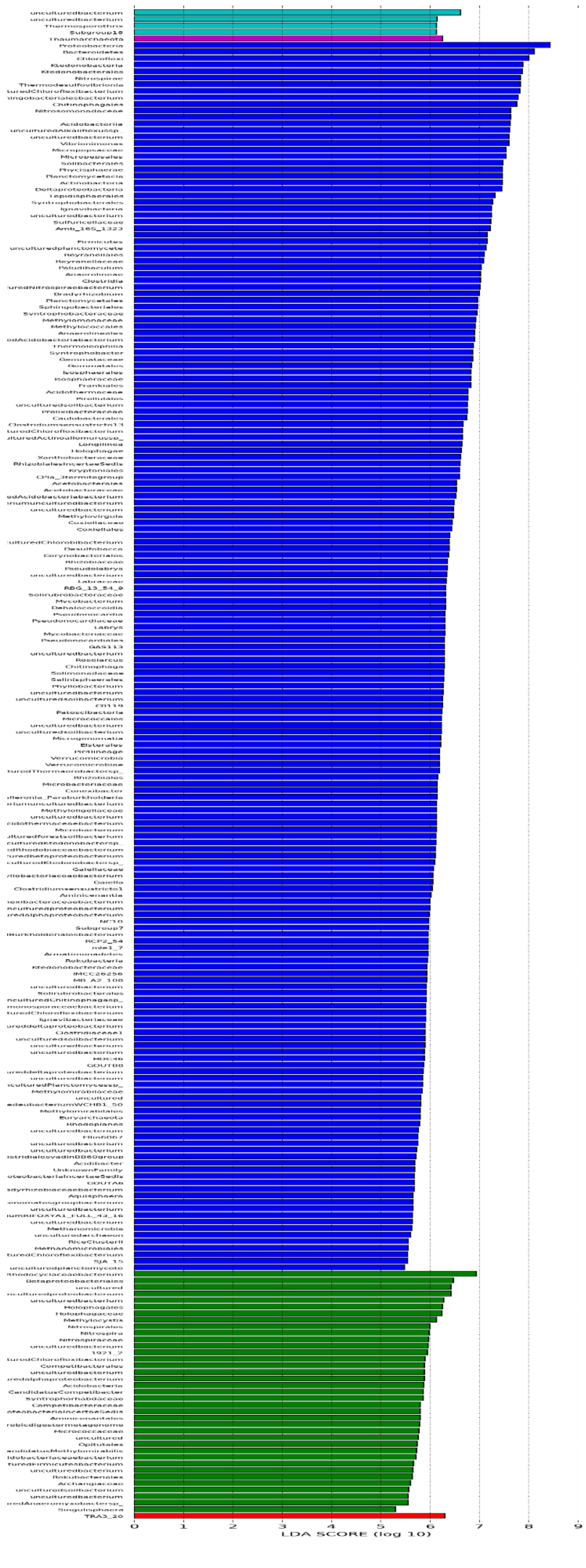

0 1 2 3 4 5 6 7 8 9
LDA SCORE (log 10)

Fig. S4. Taxa with a significant difference in 5 different mesocosms as detected by LEfSe analysis with a LDA threshold score >5 and p <0.5.
